# Investigating the Role of Free-Ranging Wild Boar (*Sus scrofa*) in the Re-Emergence of Enzootic Pneumonia in Domestic Pig Herds: A Pathological, Prevalence and Risk-Factor Study

**DOI:** 10.1371/journal.pone.0119060

**Published:** 2015-03-06

**Authors:** Mainity Batista Linhares, Luc Belloy, Francesco C. Origgi, Isabel Lechner, Helmut Segner, Marie-Pierre Ryser-Degiorgis

**Affiliations:** 1 Centre for Fish and Wildlife Health (FIWI), Vetsuisse Faculty, University of Bern, Bern, Switzerland; 2 Institut Galli-Valerio, Laboratoire d’Analyses vétérinaires, Département du Territoire et de l’Environnement, Lausanne, Switzerland; 3 Veterinary Public Health Institute, Vetsuisse Faculty, University of Bern, Bern, Switzerland; Justus-Liebeig University Giessen, GERMANY

## Abstract

Enzootic pneumonia (EP) caused by *Mycoplasma hyopneumoniae* has a significant economic impact on domestic pig production. A control program carried out from 1999 to 2003 successfully reduced disease occurrence in domestic pigs in Switzerland, but recurrent outbreaks suggested a potential role of free-ranging wild boar *(Sus scrofa)* as a source of re-infection. Since little is known on the epidemiology of EP in wild boar populations, our aims were: (1) to estimate the prevalence of *M. hyopneumoniae* infections in wild boar in Switzerland; (2) to identify risk factors for infection in wild boar; and (3) to assess whether infection in wild boar is associated with the same gross and microscopic lesions typical of EP in domestic pigs. Nasal swabs, bronchial swabs and lung samples were collected from 978 wild boar from five study areas in Switzerland between October 2011 and May 2013. Swabs were analyzed by qualitative real time PCR and a histopathological study was conducted on lung tissues. Risk factor analysis was performed using multivariable logistic regression modeling. Overall prevalence in nasal swabs was 26.2% (95% CI 23.3–29.3%) but significant geographical differences were observed. Wild boar density, occurrence of EP outbreaks in domestic pigs and young age were identified as risk factors for infection. There was a significant association between infection and lesions consistent with EP in domestic pigs. We have concluded that *M. hyopneumoniae* is widespread in the Swiss wild boar population, that the same risk factors for infection of domestic pigs also act as risk factors for infection of wild boar, and that infected wild boar develop lesions similar to those found in domestic pigs. However, based on our data and the outbreak pattern in domestic pigs, we propose that spillover from domestic pigs to wild boar is more likely than transmission from wild boar to pigs.

## Introduction

Enzootic pneumonia (EP) is one of the most important sources of disease-associated losses in swine production [1–3]. This lung disease is caused by *Mycoplasma hyopneumoniae* (Mhyop). The classical clinical presentation is a sporadic non-productive cough and retarded growth. Economic losses result from inefficient food/weight conversion and increased drug usage [4,5]. Enzootic pneumonia mainly affects mid- to late finishing pigs, though infection can be traced back to birth. The main infection route is direct contact with infected swine (sow and pen mates). Infections in previously EP-free herds can often be traced back to the introduction of subclinically infected animals into the herd [4,6,7]. Airborne transmission has also been reported and is most likely to occur within a herd [7,8]. Nevertheless, viable Mhyop have been detected up to 9.2 km from an infected herd [7–9].

The presence and severity of clinical signs in growing pigs (coughing) and the pathological findings (cranio-ventral lung lesions, histologically characterized by perivascular and peribronchiolar lymphocytic cuffing, type II alveolar pneumocyte hypertrophy and alveolar inflammation [10,11]) vary according to the disease stage. They are therefore weak indicators of Mhyop infection [11,12]. Until recently, the most commonly used diagnostic method for detecting Mhyop was a combination of serological analyses, including ELISA and immunofluorescence test [13]. Real-time polymerase chain reaction (real-time PCR) has since been developed for this pathogen and has increased the detection success of Mhyop infection (higher sensitivity and specificity of the test) [14]. Real-time PCR has a specificity of 100% in domestic pigs, with a sensitivity of 85% using bronchial swabs [14]. Sensitivity at individual level is low when using nasal swabs (47.1%) but herd-level sensitivity reaches 100% in herds including coughing pigs (average sample size of 10 pigs per herd) [15].

Enzootic pneumonia has been drastically reduced in the swine population in Switzerland through a control program carried out from 1999 to 2003 [16,17]. However, the re-emergence of EP on individual farms in the absence of obvious sources of infection, including a farm in the region of Geneva [18] where wild boar density is among the highest in Europe [19], raised the question of whether or not free-ranging wild boar could play a role in infection. Wild boar belong to the same species as domestic pigs. They share many of the same pathogens [20,21] and transmission between wild boar and pigs is possible [22]. Wild boar populations, therefore, may act as a reservoir for these pathogens [23–25]. Serological studies in free-ranging wild boar in Europe have revealed a high prevalence of antibodies against Mhyop, ranging from 21% in Slovenia and Spain to 30% in Italy and 58% in France [26–29]. In Spain, Mhyop has been detected by nested PCR in 8% of sampled lungs (BS) and 20% of nasal swabs [27], while in Italy Mhyop DNA was detected by qualitative real-time PCR in 46% lungs sampled [28]. More recently, investigations carried out in Geneva using real-time PCR on lung tissue [30] have detected a 41% prevalence of Mhyop in wild boar in the area. Overall, these data suggest that Mhyop is widespread in free-ranging wild boar populations, but the diversity of methods and materials used for prevalence estimations prevent reliable comparisons among regions. Furthermore, only two of these studies have addressed the question of risk factors for infection, and this was limited to age, sex and adjacent hunting districts [27,28]. Lung lesions resembling EP have been observed in wild boar [27,28] but the macroscopic and histologic changes in these animals have not been characterized, and the association between infection and the occurrence of lesions has not yet been clearly established in wild boar.

Overall, no comprehensive study has been performed to date to assess the role of wild boar in the epidemiology of this economically important disease of domestic livestock. The objective of this study was to investigate the epidemiology and pathology of EP in wild boar, in order to contribute to the information necessary for the control of the infection in domestic pigs. More specifically, our aims were: (1) to estimate the prevalence of Mhyop infections in wild boar from different geographical regions; (2) to identify risk factors for Mhyop infection in wild boar, considering both individual and environmental factors; and (3) to assess whether infection in wild boar is associated with the same macroscopic and histologic pathological features typical of EP in domestic pigs. We conducted a cross-sectional study and detailed pathological investigations, hypothesizing that Mhyop is widespread in Swiss wild boar but that wild boar are mostly healthy carriers, developing no to only mild lung lesions.

## Materials and Methods

### Study areas

**Fig 1:**
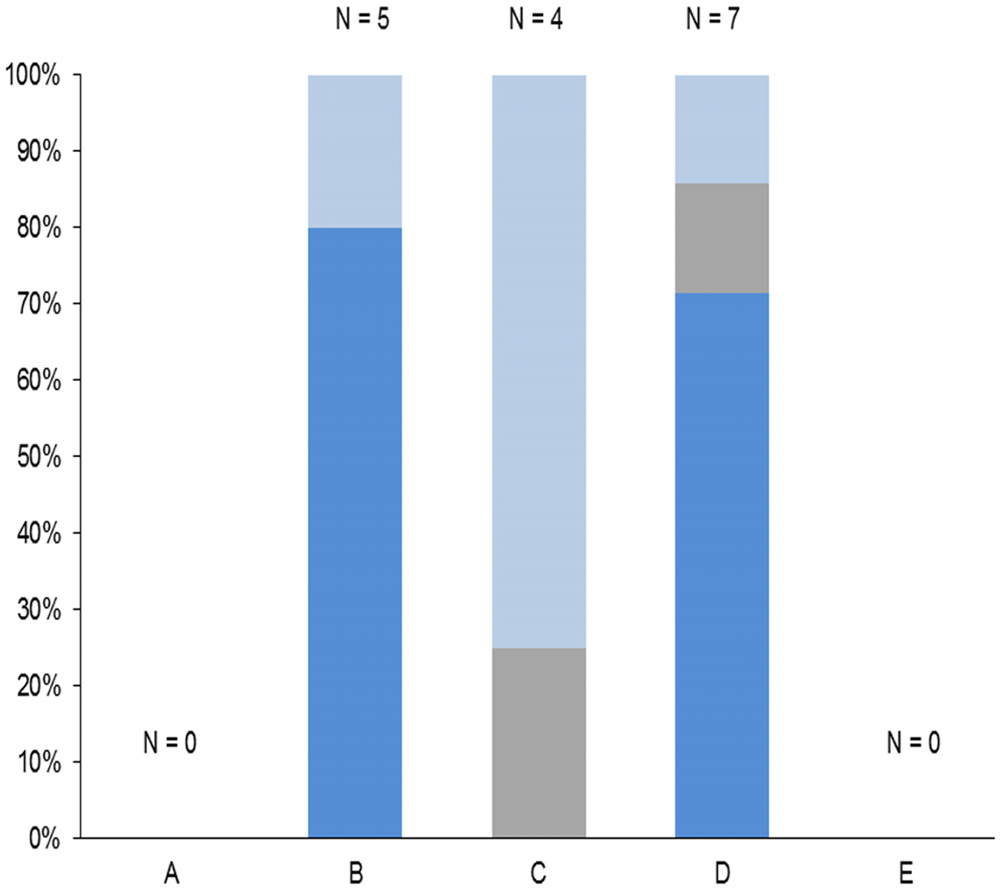
Registered outbreaks of enzootic pneumonia in domestic pigs from 2010 to 2013. The number of outbreaks (N) is indicated for each study area (units A-E) and colors indicate the source of infection. Dark blue: Domestic pig. Grey: Unknown, wild boar unlikely. Light blue: Unknown, wild boar suspected.

This study was carried out in Switzerland (41’285 km^2^). Five sampling units (A, B, C, D and E) were defined based on the following criteria: 1) wild boar density index (WBDens) or relative abundance, calculated by dividing the number of recorded dead wild boar (including hunting bag and animals found dead [31]) by the unit surface (km^2^); 2) estimated density of outdoor piggeries (OPDens), calculated by dividing the number of registered piggeries (dataset of Wu et al. [32]) by the unit surface; 3) geographical characteristics of Swiss bioregions (Federal Office for the Environment [33–35]); 4) local climate (Federal Meteorology and Climatology Department [36–38]); and 5) occurrence of EP outbreaks in the domestic pig population between 2010 and 2013 (Fig. 1, data from the cantonal veterinary offices and [39]).

**Fig 2:**
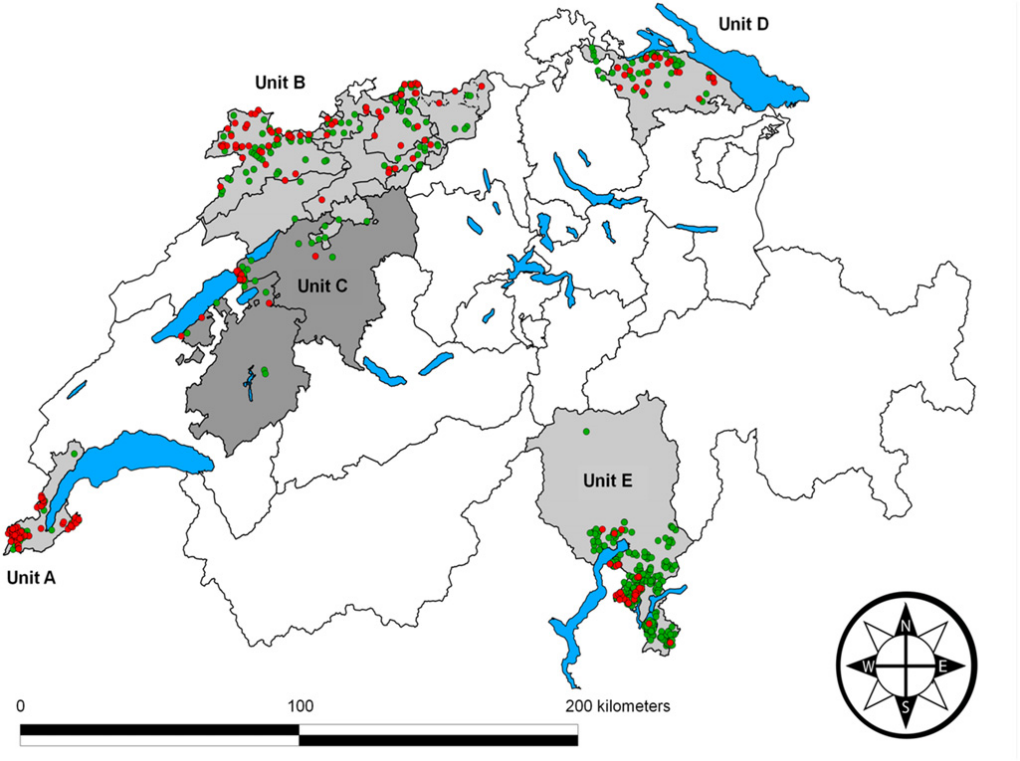
Map of Switzerland depicting the location of the study areas. The five study areas (units A-E) are indicated by shades of grey. Black lines correspond to canton borders, and blue areas are main lakes. The origin of wild boar (*Sus scrofa*) samples and their real-time PCR results are indicated by colored dots: Samples negative for *Mycoplasma hyopneumoniae* are green and positive samples are red.

The location of the five units within the country and their characteristics are indicated in Fig. 2 and Table 1, respectively. Unit A largely corresponds to the canton of Geneva, on the border of France. It is characterized by one of the highest wild boar densities in Europe (from 10.6–10.0 individuals/km^2^ [19]) and the highest WBDens in this study. Unit B lies in the heart of the Jura Mountains and covers the cantons of Jura, Basel-Land, a great part of Solothurn and smaller regions of Aargau and Bern. It borders France and Germany. Unit C lies in the center of the Swiss Plateau and covers the canton of Freiburg and a large part of Bern. Its WBDens is relatively low but unevenly distributed within the area (most wild boar being present in the north-west of the region) and the OPDens is the highest of all units. Unit D corresponds to the canton of Thurgovia and borders Germany. It has a hunting bag comparable to unit A but moderate WBDens and OPDens. Unit E corresponds to the canton of Tessin at the border with Italy. Its hunting bag is the highest of all Swiss cantons (over 1000 wild boar in each of the two last reported yearly hunting bags) and its wild boar population is therefore considered as very large despite a moderate WBDens.

**Table 1:** Study areas (geographical units A-E) and their characteristics.

Unit	Wild boar density^a^	Density of outdoor piggeries^b^	Predominant Climate					Prevalence of Mhyop^d^		Predominantly detected DNA target^e^		Outbreaks of enzootic pneumonia (year of last outbreak)^f^
			Air temperature		Humidity							
			min.-max. (°C)	OD^c^	Precipitation (mm)	Relative humidity (%)	OD^c^	%	95% CI	Target	Detection frequency(%)	
**A**	very high	very low	6.2–15.2	warm	100.1	73	dry	54.6	46.0–63.1	ABC/REP	71.0	0 (2007)
**B**	medium	low	3.9–13.9	cold	966–1118	76–79	humid to equilibrate	35.4	29.1–42.1	ABC/REP	53.8	5 (2013)
**C**	low	high	4.7–14.1	mild to cold	983–1123	76.5–77	equilibrated	20.3	11.2–32.2	ABC/REP	53.8	4 (2011)
**D**	medium	medium	4.1–12.4	mild	1085	78.5	equilibrated	35.6	24.7–47.6	ABC/REP	46.1	7 (2012)
**E**	medium	very low	6.7–24.5	warm	1542	69.6	disequilibrated (dry and humid seasons and or areas)	8.4	5.8–11.9	ABC only	63.3	0

^a^ Wild boar density index categories correspond to the following estimations: very high = 1.65 dead individuals/km^2^ per year, medium = 0.40–0.54 and low = 0.04.

^b^ Density of outdoor piggeries correspond to the following estimations: high = 0.31 outdoor piggeries/km^2^, medium = 0.19, low = 0.08 and very low = 0.01.

^c^ OD = Official definition. The climatic characteristics of the five geographical units are based on measurements of the Swiss Federal Office of the Environment, Transport, Energy and Communication, Federal Meteorology and Climatology Department, Federal Agronomy Office.

^d^ Prevalence (%) and 95% confidence interval (95% CI) of *Mycoplasma hyopneumoniae* in wild boar nasal swabs (PCR analysis) in 2011–2013 (this study).

^e^ predominant *M*. *hyopneumoniae* type (ABC only, REP only, ABC/REP).

^f^ Number of recorded outbreaks of enzootic pneumonia in domestic pigs in 2010–2013.

### Wild boar samples

The required sample size for prevalence estimation was calculated with the WinEpiscope 2.0 software package for an expected prevalence of 50%, with a confidence level of 95% and an accepted error of 5%. We aimed at a total of 150 animals per unit and year with an even age and sex distribution among units. Following a preliminary evaluation of different sampling materials [40], we chose to collect nasal swabs for the prevalence study. Additionally, we aimed at collecting at least 60 lungs with an even distribution of lesion categories (see definitions below).

Sampling was carried out by game-wardens, hunters, or veterinarians from October 2011 to May 2013 (two consecutive hunting seasons). Hunting seasons (HS) were defined as: HS1, from 1^st^ October 2011 to 15^th^ July 2012; and HS2, from 16^th^ July 2012 until 1^st^ May 2013. Date of sampling, geographical coordinates, biological data (age, sex, weight and body condition), and information on presence of an outdoor piggery including the estimated distance between the piggery and the shooting place (OPDist) were collected for each animal using a standardized form. Based on wild boar behavior [41–43], a former risk factor study in Swiss wild boar [22] and a study on the role of infectious aerosol on transmission of diseases in swine [44], two categories were defined: category 1 (OPDist ≤ 1000m) and category 2 (OPDist >1000m).

A total of 961 nasal swabs and 122 lungs were collected from 978 wild boar. Lungs from nine wild boar sampled in 2010 were also included in the pathological study. Samples which were not taken by the staff of the Centre for Fish and Wildlife Health (FIWI, Bern, Switzerland) were shipped by priority mail immediately after collection. Samples originated from 510 females and 440 males. Information on sex was missing for 28 animals. Age classification was based on the animal weight and coat color [19,32]: piglets (striped, <20 kg, 4–6 mo); juveniles (reddish, 20–40 kg, 6–12 mo); subadults (black, 41–60 kg, 12–24 mo); and adults (black or silver, large size, >60 kg; >24 mo). Information on age was missing for nine animals. Body condition was estimated as poor to moderate (later referred to as “poor”) or good to very good (later referred to as “good”) based on muscle mass and fat deposits. Information on body condition was missing for 174 animals.

### Genomic DNA and real-time PCR

Bronchial swabs were taken from wild boar lungs upon arrival at the laboratory. Both nasal and bronchial swabs were immediately processed for total DNA extraction by soaking them in a lysis buffer according to an established protocol [14]. The total DNA suspension was then stored at-20°C for a maximum of one year. Samples were analyzed by real-time PCR following the protocol of Kuhnert et al. [30]. Due to the initial observation of a high level of PCR-inhibition (data not shown), samples were systematically eluted and the assays for the two targets were run in parallel as previously performed [14,15] and not as multiplex PCR. The two targets included in the PCR protocol were: REP (repeated element MHYP1–03–950; accession no. AF004388) and ABC (I-141 DNA fragment encoding a putative ABC transporter; accession no. U02537). Based on field studies with nasal swabs in domestic pigs (established PCR protocol with a total of 50 cycles (CT) per run [15]) and observed differences in sensitivity depending on the machine equipment (ring trial, data not shown), we performed a total of 55 CT per run with the ABI 7500 Real-Time PCR System (Applied Biosystems, Foster City, CA, USA). Samples were considered positive when a clear exponential curve crossed the threshold bar at 53 CT at the latest. Quantitative PCR data were not used, because to date no data on minimum detectable amount of Mhyop are available for this protocol in samples harvested from nostrils, and because the variable initial lysate volumes due to sample contamination and the elution procedure mentioned above prevented the generation of reliable quantitative information.

### Pathological Assessment of Lungs

**Fig 3:**
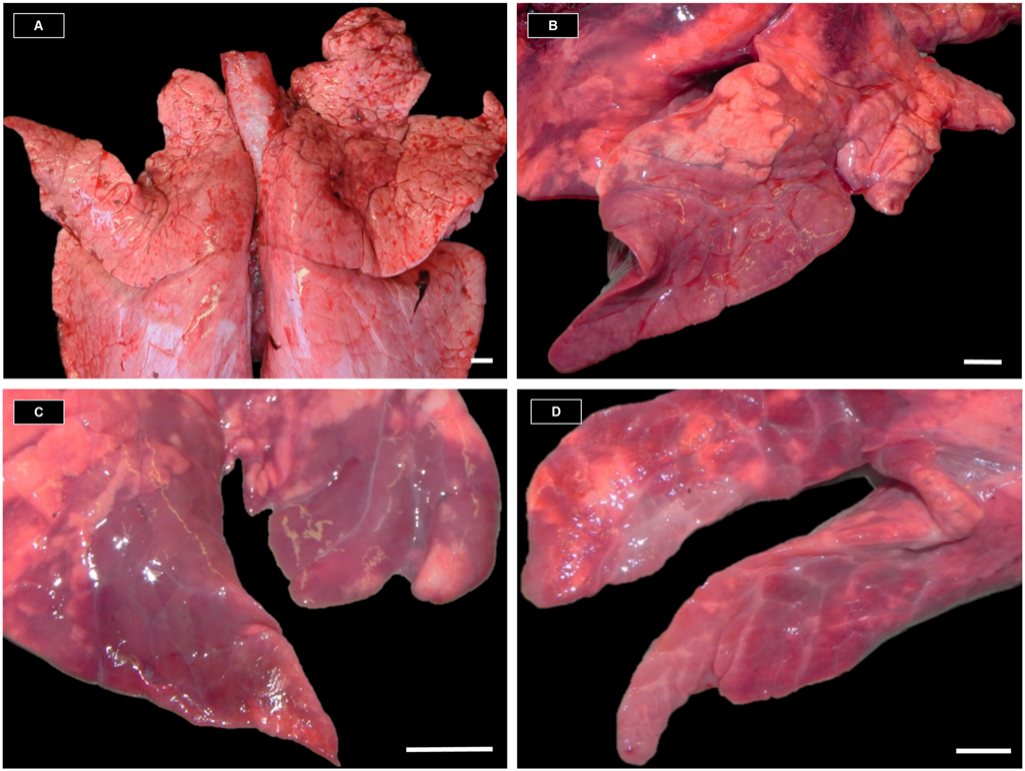
Wild boar lungs with and without macroscopic enzootic pneumonia-like lesions (MaEPL). A: Lung without MaEPL. B: Lung with early type MaEPL (red to dark red multilobular to coalescing consolidated areas affecting less than 50% of the lobe). C: Lung with late type MaEPL (multilobular to coalescing pale purple to grayish areas, showing signs of retraction of parenchyma and atelectasis). D: Lung with late type MaEPL (same as C but with more pronounced fibrosis). Measurement bars = 1cm.

Lungs were assessed to investigate (1) whether EP-like pathological lung lesions occur in wild boar, and if yes, whether they follow the same pattern as in domestic pigs; and (2) whether EP-like lesions in wild boar are associated with the presence of Mhyop DNA in bronchial and nasal swabs. We developed a standardized protocol based on former descriptions of EP lesions in lung of domestic pigs [7,12,45,46]. We then categorized the observed lung lesions in wild boar using this protocol. Lungs were photographed and evaluated macroscopically by qualified veterinary staff immediately after they were collected or received, to record lesions compatible with enzootic pneumonia (EP-like lesions). Macroscopic EP-like lesions (MaEPL) were defined as multilobular to coalescing, red to dark red to violet to grayish, mildly to severely consolidated areas of the cranial lung lobes (apical and cardiac) [7,12,45,46] and three categories of lesions were established: 1) no MaEPL; 2) early type (subacute) MaEPL, i.e. red to dark consolidations; 3) late type (chronic and end-stage) MaEPL, i.e. pale-greyish consolidations, with additional fibrotic scars in end-stage (Fig. 3). Distribution patterns were recorded separately and classified as: 1) multilobular; 2) multilobular to coalescing, affecting less than 50% of the lobe; 3) multilobular to coalescing, affecting approximately 50% of the lobe; and 4) affecting over 50% of the lobe. Additionally, we documented whether or not purulent exudate could be expressed from the cut surface of the lung.

Selection criteria for histopathological examination of lungs were: no or only mild tissue damage due to the killing shot, and absence of lung lesions or presence of MaEPL. Whenever possible, all four cranial lobes (both apical and both cardiac lobes) were sampled. Specimens were fixed in 10% buffered formalin, embedded in paraffin, sectioned at 4μm and mounted on positively charged glass slides (Super-Frost, MenzelGläser, Germany), stained with haematoxylin and eosin (H&E) according to the standard protocol in use in the Department of Infectious Diseases and Pathobiology of the University of Bern, and analyzed by light microscopy. For histological examination, we developed a protocol for standardized semi-quantitative assessment of microscopic EP lesions based on published descriptions in domestic pigs [7,10,47,48]. Airways and alveolar parenchyma (together with the interstitium) were assessed as separate components but following similar schemes. We assessed the severity of inflammation by estimating the number of detectable lymphoid follicles in the hyperplastic bronchial associated lymphoid tissue (BALT) (Table 2).

**Table 2:** Selected features for the histological assessment of wild boar lungs.

Feature	Localization	Measure
**Infiltration with inflammatory cells**	**Bronchi, bronchioles** ^**a**^	Most abundant cell-type at 20x PF^b^ in three selected fields: lymphocytes, histiocytes, plasma cells, eosinophils, neutrophils
	**Interstitium and/or intra-alveolar**	Cellularity and estimated proportion of affected parenchyma (%): mild (1–30%), moderate (31–60%), high (61–100%)
	**Perivascular**	Diameter at 10x PF in three randomly selected fields: 1x AL ^C^, 2x AL, 3x AL, 4x AL, 5x AL, >5x AL
**BALT hyperplasia**		Mean number of lymphoid follicles per field at 4x PF in three randomly selected field
**Nematode**		Absence / presence at 20 PF in three selected fields. Association with granuloma
**Edema**	**Alveoli**	Absence / presence at 20 PF in three selected fields
**Bacteria and/or fungi**		Presence at 20 to 40 PF in three selected fields

^a^ The lumen, mucosa and peripheral tissue were assessed separately for each selected structure.

^b^ PF = Power field. ^C^ AL = Microscope arrow length (Nikon model Eclipse E 400).

### Data Analysis and Statistics

Data management was conducted with Microsoft Excel^©^ (Microsoft Corporation, Redmond, Washington, USA). Basic statistical calculations were performed with NCSS 2010 Statistical Software (J. L. Hintze, Kaysville, Utah, USA), following transfer of the spread sheets from Microsoft Excel^©^. Prevalence was calculated assuming a test specificity and sensitivity of 100%. Only interpretable PCR results (N = 849) were included in the study. The Wilcoxon Signed-Rank Test and the Kappa-Test were applied for comparing PCR results obtained with nasal and bronchial swabs (96 wild boar). The Fisher’s Exact Test and the Wilcoxon Signed-Rank Test were used for assessing association (p-value < 0.05) between MaEPL, histopathological findings and PCR results obtained with bronchial swabs (106 wild boar). The Fisher’s Exact Test was also used to assess association between poor body condition and the presence of macroscopic and histologic lung lesions (features found to be associated with PCR-positive bronchial swabs, see results) and between poor body condition and PCR-positive nasal and bronchial swabs. Maps were designed using the Quantum GIS software, version 1.8.0 Lisboa (©OSGeo Project).

Age class, sex, hunting season, geographical unit and associated characteristics, as well as OPDist were considered as possible risk factors for infection with Mhyop (based on data in domestic pigs [1,5,49]) and were therefore selected as independent variables for the model. For further analysis of the risk factors, a two-stage logistic regression was performed using StataCorp. 2012 (StataCorp. 2011. *Stata Statistical Software*: *Release 12*. College Station, TX: StataCorp LP). The dependent (outcome) variable was binary and indicated whether the animal was infected with EP or not, based on PCR result of nasal swabs. First, risk factors with a p-value < 0.2 were identified through a univariable logistic regression model and then considered as candidates for the multivariable model. A Spearman’s rank correlation matrix was generated to identify strongly correlated candidate variables. If strong correlation (Spearman’s rank correlation coefficient > ± 0.4) was observed, variables for the full model were selected according to their biological relevance and estimated importance for the study questions. A manual backward elimination procedure was performed with a cut-off level at p-value < 0.05, determined by the Wald Test. If the regression coefficient of the remaining variables changed more than 20% after the removal of a non-significant variable, it was considered as a confounder. The candidate variables were all categorical and for an optimal interpretation of odds ratios (OR), the category with the lowest Mhyop prevalence of each variable was selected as the baseline category in the model. After getting to the final model, the variable unit was added as a random effect to check for its potential influence as a cluster variable and the intraclass correlation coefficient (ICC) was estimated. To compare the fit of the models, the Akaike Information Criterion (AIC) was estimated for the two model approaches.

### Ethics statement

All samples originated from dead wildlife, which was either hunted (N = 735), legally shot for population control (N = 216) or due to severe debilitation (N = 4), or found dead (N = 8). According to Switzerland’s legislation (922.0 hunting law and 455 animal protection law, including legislation on animal experimentation;www.admin.ch), no ethical approval or permit for animal experimentation was required.

## Results

### Diagnostic Performance with bronchial swabs and nasal swabs

Analyses of samples from 96 wild boar from which both bronchial and nasal swabs were obtained, revealed a higher detection percentage with bronchial (57.2%, N = 55/96) than nasal swabs (37.5%, N = 36/96), i.e. the estimated prevalence significantly differed among sampling materials (p = 0.0091). While 25 animals tested PCR-positive with bronchial but negative with nasal swabs, the contrary occurred for six animals, and the obtained kappa-value of 0.39 suggested a low agreement between the results generated with these two sampling materials. Regarding the PCR reaction, 95% of the samples showed a CT-value ≤ 53 but the occurrence of the curves was significantly delayed of 5–6 CT with nasal compared to bronchial swabs (p-value < 0.0001).

### Detected PCR Targets

**Fig 4:**
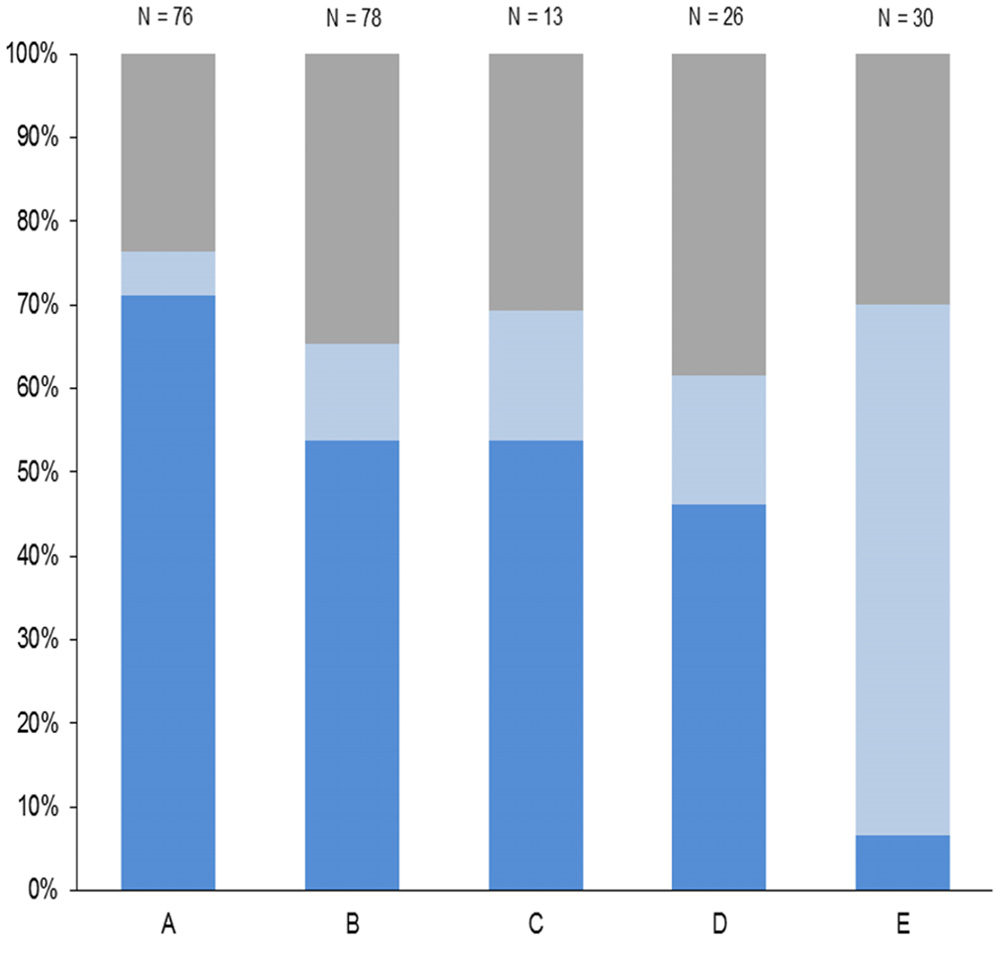
Detected target groups of *Mycoplasma hyopneumonia* types in the five study areas. Data refer to PCR-positive nasal swabs from wild boar. Study areas: units A-E. Dark blue: ABC/REP type. Light blue: ABC-only type. Grey: REP-only type.

Both PCR targets (ABC and REP) were amplified in about half (52.4%, N = 117/223) of the real-time PCR-positive samples of nasal swabs. From all positive nasal swabs, 30.4% (N = 68/223) was detected only by the REP target and 17.0% (N = 38/223) only by the ABC target. All three Mhyop types (ABC/REP, ABC only, and REP only) were present in samples from each geographical unit but in different proportions. While in the northern wild boar population (units A-D) both targets were usually detected within a sample (59.5%, N = 115/193), in unit E the majority of the PCR-positive wild boar reacted only to the ABC target (Fig. 4, p-values < 0.0001).

### Pathological Assessment of Wild Boar Lungs

Early or late type macroscopic EP-like lesions (MaEPL) were recorded in 52 of 106 wild boar lungs. See Table 3 for a detailed classification. Lungs with late type MaEPL presented significantly more affected areas than lungs with early type MaEPL. Among late type MaEPL, end-stage lesions with scars were observed in one animal only. The presence of MaEPL was associated with a positive PCR reaction for Mhyop in bronchial swabs (p-value = 0.0175).

**Table 3:** Distribution patterns of 52 lungs with macroscopic lesions considered typical for enzootic pneumonia (“EP-like”, early and late types) and PCR results.

	Early type	Late type^a^
**Multilobular**	13 (1)^b^	3
**Multilobular to coalescing; < 50% of each lobe affected**	9 (1)	6
**Multilobular to coalescing; 50% of at least one lobe affected**	3	12 (2)
**Multilobular to coalescing; > 50% of at least one lobe affected**	1	5 (1)
**PCR** ^**c**^ **positive/total**	18/26	20/26

^a^ Late-type lesions were significantly associated with larger affected lung areas (multilobular coalescing, ≥ 50% of infected lobe), p-value = 0.0005.

^b^ Numbers in brackets correspond to the specific subgroup of lungs which had exudate oozing from the airways.

^c^ On bronchial swabs.

**Fig 5:**
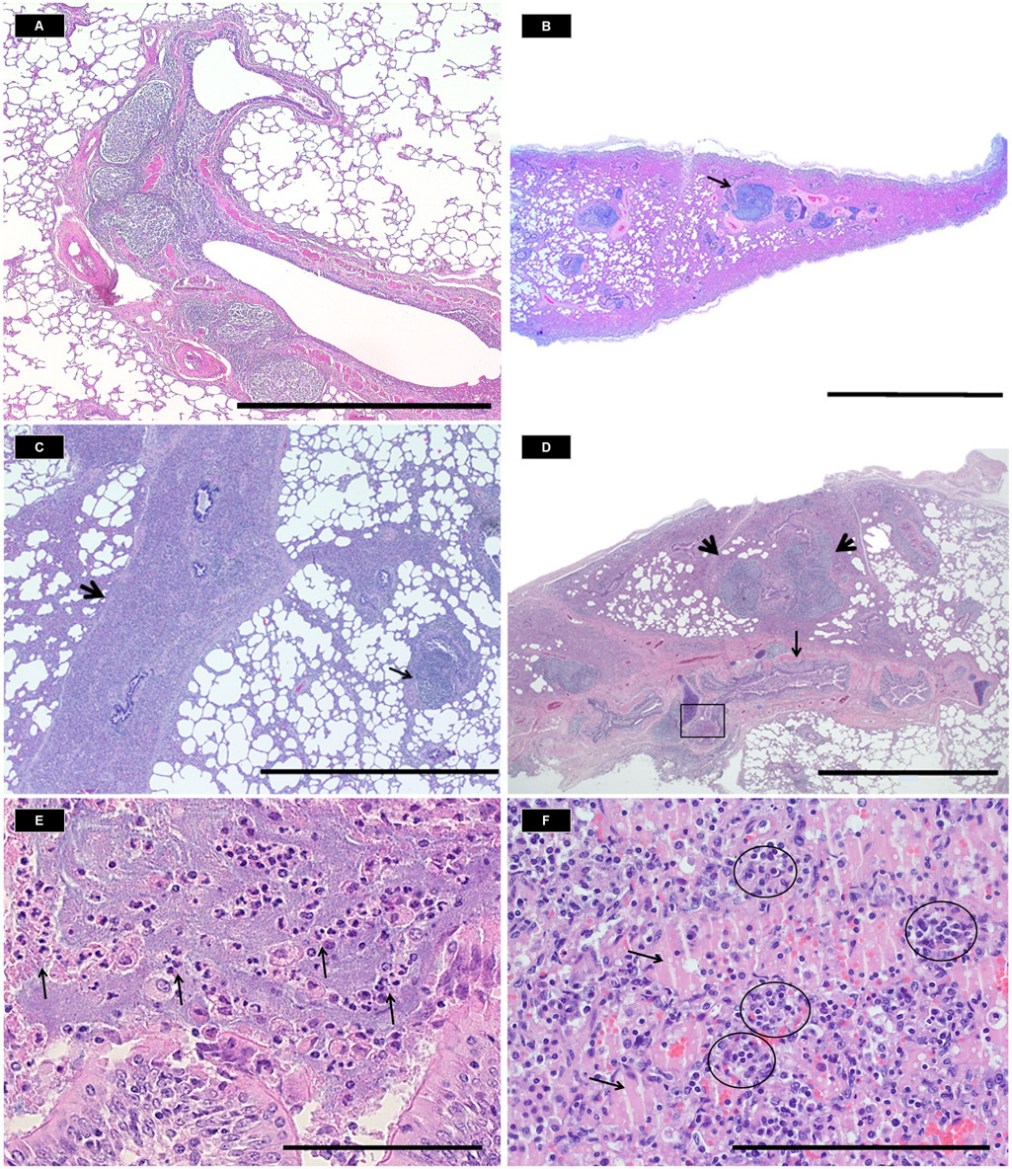
Histological lesions associated with *Mycoplasma hyopneumoniae* infection and their distribution in wild boar lungs. A: Lung without macroscopic enzootic pneumonia-like lesions (MaEPL) with moderate multifocal BALT hyperplasia along a bronchiole (measurement bar = 500nm). B: Lung with early type MaEPL and mild to moderate multifocal BALT hyperplasia (black arrow) and diffuse moderate thickening of the interalveolar septa (broncho-interstitial pneumonia). Marginal subpleural atelectasis is considered an artefact (measurement bar = 50mm). C: Lung with early type MaEPL and severe infiltration of mononuclear cells expanding the interlobular septum (thick black arrow) and compressing the regional airways. A discrete hyperplastic lymphoid follicle is also present (thin black arrow, measurement bar = 2mm). D: Lung with late type MaEPL and moderate to severe multifocal BALT hyperplasia (thick arrows), moderate infiltration of the submucosa of the airways (thin black arrow) and mild to moderate multifocal thickening of the interalveolar septa by infiltrating inflammatory cells (measurement bar = 500nm). E: Magnification (20x) of the framed area in D (inset). Intraluminal collection of neutrophils (thin black arrows, measurement bar = 20μm). F: Lung with late type MaEPL and diffuse intraalveolar collection of amorphous eosinophilic material (edema, black arrows) with mild to moderate multifocal lympho-plasmacytic clusters in the interstitium (black circles). Few numbers of mononuclear cells are also observed in the alveolar spaces along with free erythrocytes (hemorrhages, gunshot-related, measurement bar = 20μm).

Histological observations are summarized in Table 4. Six of 14 selected histopathological features were significantly associated with a positive PCR reaction for Mhyop in bronchial swabs; the most frequently observed was BALT hyperplasia together with lymphoid follicle activation (p-value = 0.0136), followed by plasma cell clusters in the interstitium (p-values < 0.0001), neutrophilic infiltration of the bronchi and bronchioles (both the lumen and the airway wall, p-value < 0.0001), intra-alveolar edema (p-value = 0.0124) and neutrophilic infiltration of the alveolar parenchyma and/or the interstitium (p-value = 0.0084). Additionally, scattered interstitial histiocytes and lymphocytes were commonly observed, tending to be more frequent in PCR-positive than PCR-negative lungs (p-value ≥ 0.161). Histological features associated with PCR-positive results were found in animals with MaEPL as well as without, but their frequency and/or severity generally increased with the chronicity of MaEPL (Table 5, Fig. 5).

**Table 4:** Frequency and distribution pattern of recorded histological features in wild boar lungs.

	Bronchi and bronchioles		Alveolar parenchyma and/or interstitium	
**Histological features**	PCR-positive N (%)	PCR-negative N (%)	PCR-positive N (%)	PCR-negative N (%)
**BALT hyperplasia** ^**a**^	53 (50.0)	24 (22.6)	-	-
**Infiltration of histiocytes**	44 (41.5)	24 (22.6)	57 (53.7)	35 (33.0)
**Infiltration of lymphocytes**	40 (37.7)	23 (21.6)	31 (29.2)	26 (24.5)
**Infiltration of plasma cells**	22 (20.7)	7 (6.6)	27 (25.4)	2 (1.8)
**Infiltration of neutrophils**	17 (16.0)	0	13 (12.2)	1 (0.9)
**Infiltration of eosinophils** ^**b**^	33 (31.1)	24 (22.6)	9 (8.4)	12 (11.3)
**Perivascular infiltration**	-	-	44 (41.5)	25 (23.5)
**Intralesional parasites**	21 (19.8)	16 (15.0)	24 (22.6)	18 (16.9)
**Intralesional bacteria or fungal organisms**	0	0	0	0
**Intraalveolar edema** ^**c**^	-	-	22 (20.7)	5 (4.7)
**Mean number of lymphoid follicles in hyperplastic BALT**	2.8	1.1	-	-
**Cellularity of cut section (%)**	-	-	Low (37.7), Moderate (44.3), High (17.9)	
**Overall distribution pattern of infiltrate (%)**	Focal (5.6%), Multifocal (87.7%), Diffuse (1.8%)		Multifocal (89.6%), Focal (1.8%), Diffuse (8.4%)	

^a^ BALT hyperplasia with active follicles was not associated with the presence of parasites (p-value = 0.3697).

^b^ Eosinophil infiltration in the airways was associated with the presence of intralesional parasites (p-value = 0.0002).

^c^ Intra-alveolar edema was associated with plasma cell clusters in the interstitium (p-value = 0.0004), parasites (p-value = 0.0467) and neutrophils in the alveolar parenchyma and/or interstitium (p-value = 0.0467). Observations are classified according to the concerned anatomic structure and the PCR results obtained with bronchial swabs. A total of 106 lungs were examined.

**Table 5:** Histological characterization of wild boar lungs classified as early type, late type and without macroscopic EP-like lesions.

	Macroscopic EP-like lesions		
**Lung histopathology**	None	Early type	Late type
**Neutrophilic infiltration in the airway**	Rarely (9%)	Occasionally (16%)	Mostly (60%)
**Neutrophilic collections into the alveolar parenchyma and/or infiltration of the interstitium**	Occasionally (14%)	Occasionally (22%)	Commonly (30%)
**Plasma cell clusters in the interstitium**	Commonly (52%)	Commonly (33%)	Commonly (50%)
**BALT hyperplasia**	Mostly (80%)	Always (94%)	Always (95%)
**Mean of activated follicles (variation)**	1.3 (0–13)	1.9 (0–7)	2.3 (0–6.6)
**Intra-alveolar edema**	Occasionally (26%)	Occasionally (14%)	Commonly (57%)
**Total**	21	18	20

A total of 106 wild boar lungs were examined. Numbers in brackets indicate the percentage of lungs affected in each category. Only features with significant association with positive PCR results in bronchial swabs are indicated. Significant differences among the three categories of macroscopic lesions were: 1) more frequent BALT hyperplasia and lymphoid follicle formation in lungs with macroscopic lesions than without (both types pooled; p-value = 0.0023); 2) higher number of lymphoid follicles in lungs with late type macroscopic lesions than without (p-value = 0.0056); 3) more frequent neutrophilic infiltration in the airways in lungs with late type lesions than in those with early type or no lesions (p-value ≤ 0.0089); 4) more frequent intraalveolar edema in lungs with late than early type lesions (p-value = 0.0448).

**Fig 6:**
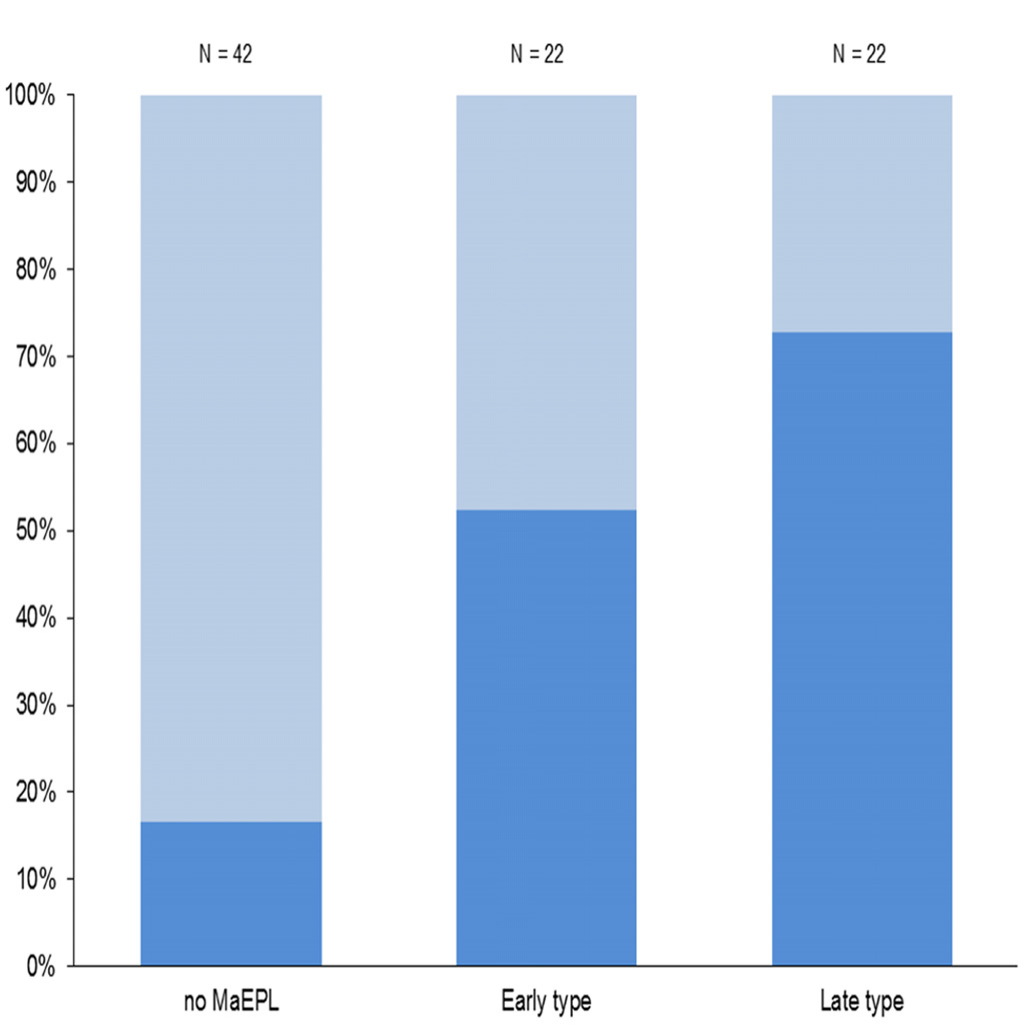
Relationship between PCR results and the absence/presence of macroscopic enzootic pneumonia-like lesions in wild boar. PCR results refer to data obtained with nasal swabs. MaEPL: Macroscopic enzootic pneumonia-like lesions (of early and late type) detected in wild boar lungs. Dark blue: PCR-positive samples. Light blue: PCR-negative samples.

Similarly, PCR-positive nasal swabs were associated with both MaEPL and typical histological features as listed in Table 5 (p-values ≤ 0.0097). Furthermore, nasal swabs tested positive more frequently in animals with late than early type MaEPL (16/22 and 11/21 individuals, respectively; Fig. 6) but this difference was not significant.

The occurrence of EP-like lesions in PCR-positive animals (bronchial swabs) seemed to decrease with age (from 72% MaEPL in juveniles to 60% in subadults and 44% in adults, and from 93% typical histological lesions in juveniles to 80% in subadults and 81% adults) but these differences among age classes were not significant (p-value ≥ 0.1068). Comparison with piglets was not carried out due to small sample size (N = 4). We found no association between poor body condition and the presence of macroscopic or histologic lung lesions (p-value ≥ 0.0724) or between poor body condition and positive PCR results (both from bronchial and nasal swabs, p-value ≥ 0.0971).

### Prevalence Study

The overall prevalence of Mhyop infection in wild boar in Switzerland was estimated at 26.2% (95% confidence interval CI 23.3–29.3%) based on real-time PCR analysis of nasal swabs. Prevalence at the unit level is given in Table 1. There was a significant difference between the northern wild boar population (units A-D; 38.9%, 95% CI 34.5–43.3) and the southern population (unit E; 8.4%, 95% CI 5.8–11.9%; p-value < 0.0001). Unit A had the highest prevalence (54.6%) and unit E the lowest, both being significantly different from all other units (p-values ≤ 0.0128).

The overall prevalence in the first hunting season (32.8%, 95% CI 27.0–36.9%) was significantly higher than in the second season (23.4%, 95% CI 20.1–27.1%; p-value = 0.0063). At unit level, there was also a prevalence decrease in unit E (p-value = 0.0313) while prevalence remained stable in unit C (p-value = 1) and differences in other units were not significant: decrease in unit A (p-value = 0.3724) and D (p-value = 0.5806), and increase in unit B (p-value = 0.1207; see S1 Table).

Piglets (18.1%, 95% CI 9.0–30.9) and adults (27.9%, 95% CI 22.3–34.1) were less frequently infected than juveniles (35.3%, 95% CI 29.7–41.2, p-values ≤ 0.0122). Adults were also less frequently infected than subadults (36.9%, 95% CI 27.1–47.6, p-values ≤ 0.0035). No significant difference in prevalence was found among sexes.

### Risk factor analysis

The correlation matrix of the potential risk factors indicated that EP outbreaks in domestic pigs were strongly correlated with both outdoor piggery density (OPDens) and temperature (correlation coefficient +0.96 and-0.96, respectively), and that OPDens was also correlated with temperature (-0.85). We selected “EP outbreak in domestic pigs” as the most relevant of the correlated variables because: (1) the main study question concerned the epidemiological role of wild boar in the re-emergence of EP in domestic pigs; (2) the association between EP occurrence and both high piggery densities and cold weather is well-known [2,17,50]; and (3) the apparent correlation between piggery density and cold weather is likely due to the rare occurrence of piggeries in parts of the country with a milder climate.

Therefore, the following variables were included in the logistic regression model: sex, age class, hunting season, distance to the next outdoor piggery (OPDist), estimated wild boar density (WBDens) and “EP outbreak in domestic pigs”. No confounding variables were identified during the backward elimination process. Age class, hunting season, “EP outbreak in domestic pigs” and “WBDens” were identified as significant risk factors for the occurrence of Mhyop in the wild boar population. In contrast, OPDist and sex were eliminated from the model (p-values ≥ 0.387). The AIC of the model was 829.32. After adding the geographical unit to the model as a random effect, the AIC was 831.32 and the ICC was clearly non-significant (rho ≤ 0.001). Due to the higher AIC value and the non-significant ICC in the random-effect model, the model without unit as random effect was selected as the final model. Factors significantly associated with a high prevalence of Mhyop and the corresponding odds ratio (OR) of the final model are presented in Table 6.

**Table 6:** Identified risk factors for *Mycoplasma hyopneumoniae* infection in Swiss wild boar.

Risk factor	Subcategory	OR	p-value	95% CI
**Age class (Baseline Adults)**	Piglets	1.53	0.301	0.68–3.46
	Juveniles	2.30	0.000	1.48–3.56
	Subadults	1.68	0.028	1.05–2.67
**Hunting Season (HS) (Baseline HS2)**	HS1	1.74	0.003	1.20–2.51
**Wild boar density (Baseline Low)**	Medium	2.22	0.019	1.14–4.33
	Very high	26.10	0.000	11.24–60.57
**EP outbreaks** ^**a**^ **(Baseline “No”)**	Yes	5.67	0.000	3.61–8.91

^a^ Outbreaks registered between 2010 and 2013. Final multivariable logistic regression model of risk factors in a study performed in 2011–2013. Significant associations with occurrence of *M*. *hyopneumoniae* in wild boar are expressed by odds ratios (OR) and respective 95% confidence intervals (95% CI). The total number of observations was 849. No confounding variables were identified.

## Discussion

This study compared the diagnostic performance of nasal and bronchial swabs for detection of Mhyop in wild boar, documented the occurrence and typical features of both macroscopic and histologic lung lesions associated with infection, estimated the prevalence of Mhyop in free-ranging wild boar from different geographical regions, and identified risk factors for infection. To our knowledge, it is the first time that such a comprehensive study has been performed on EP in wild boar, enabling a better evaluation of the role of wildlife in the epidemiology of this economically important disease.

### Detection of *M*. *hyopneumoniae* in Lungs and Nasal Swabs

Lungs have been reported as the organ of choice for detection of Mhyop in domestic pigs [51,52]. This may be due to the amount of detectable DNA, which is larger in lung samples than nasal swabs [53]. In this study, detection success was indeed higher in bronchial than nasal swabs. However, a previous study in wild boar reported a higher success with nasal than with bronchial swabs [27], and we found no significant difference between prevalence estimated with either method in our pilot study [40]. The origin of these variations is unclear but the use of a different PCR protocol (nPCR [27], which is generally considered less specific and more susceptible to contamination than real time PCR [14,54,55]) and the obtained sample sizes may have played a role. Nevertheless, the use of nasal swabs has two advantages: it enables the achievement of a higher sample size through better compliance of field partners such as hunters, because sampling and shipping is much easier; and it is associated with lower shipping costs than lung samples, which make a considerable budget difference when aiming at a large sample size. Furthermore, we consider that nasal swabs represent a good choice in the frame of this study, because the main goal was to assess the role of wild boar in the recurrence of EP in domestic pig herds. Infection with Mhyop occurs mainly through nose-to-nose contact or among animals in close proximity to each other [6,56–58]. It is likely that we are identifying the animals shedding the bacteria through their upper airways, i.e. the most epidemiologically significant animals, when we detect Mhyop in nasal swabs.

We observed a delay in the amplification signal of the PCR with nasal swabs compared to bronchial swabs. This difference in the PCR reaction is likely due to the above-mentioned difference in DNA quantity between the two types of swabs. However, since in some cases nasal swabs turned out to be PCR-positive while bronchial swabs were negative, the varying detection success in either material may also be related to different stages of infection. In domestic pigs, Ruiz et al. [59] proposed that nasal swabs test positive by PCR mainly at the beginning of the infection process and in the latest phase of the infection, while they are mostly negative during the period in between. Pieters et al. [60] indeed reported that shedding through the nose may start before the onset of coughing, which is usually observed in the presence of lung lesions [12,61,62]. Furthermore, pigs may remain infectious for other pigs up to 200 days post inoculation despite apparent full recovery from clinical disease and negative PCR in lung tissues [60]. Although nasal swabs were not collected in the late phase of this study, shedding through the nose was the likely route of bacterial spread to other animals, since fomites play a minimal role in transmission of Mhyop [63].

In our study, positive PCR results obtained with either bronchial or nasal swabs were associated with lung lesions (both macroscopic and histologic). This suggests that the probability of detecting Mhyop in both kinds of samples is influenced by the presence of tissue damage. Two further observations indicate a possible relationship between the presence or severity of the lesions and the amount of bacteria which are shed: 1) More wild boar with MaEPL tested positive in nasal swabs than wild boar with only microscopic lesions, and 2) the percentage of positive nasal swabs increased with chronicity of the lesions, which in turn was associated with more extensive lesions. A parallel increase of the amount of detectable mycoplasma DNA and of the presence/severity of lesions has been reported for other *Mycoplasma* spp. in other host species, such as in *M*. *bovis* in cattle and *M*. *conjunctivae* in Alpine chamois (*Rupicapra rupicapra rupicapra*) and ibex (*Capra ibex ibex*) [64–67], as well as for other infectious agents such as mycobacteria [68–71]. One factor that may play a role in this process is strain virulence. It has indeed been reported that virulent strains of Mhyop not only lead to more pronounced disease signs (lung lesions and coughing) [61,62,72] but that they are also characterized by a higher mycoplasma proliferation rate in lung tissue [51,53].

### PCR Protocol and DNA Targets

The PCR protocol selected for this study is used as a diagnostic method in pig herds with disease signs [15], and the two nucleotide targets are considered to be well conserved [8,73,74]. Moreover, the protocol has shown a very high sensitivity and specificity for strains identified in field studies both in Switzerland and North America [14,55]. Therefore, we believe that our PCR protocol was appropriate to detect strains relevant for EP outbreaks in domestic pigs. Different combinations of the two targets were observed in the material analyzed in this study. This is in agreement with former investigations in Switzerland, which have identified the same three major types of Mhyop circulating in domestic pigs and in wild boar [14,30]: the ABC-only, the ABC/REP and the REP-only type.

### Lung Lesions

Wild boar infected with Mhyop typically presented a mild to moderate lympho-histiocytic broncho-interstitial pneumonia affecting mostly the cranio-ventral portion of the lung and characterized by BALT hyperplasia, and scattered plasma cell clusters; with increasing chronicity of the inflammation process, neutrophil collections (patchy bronchopneumonia) and intra-alveolar edema were also observed. This is consistent with EP in domestic pigs [13,45,47,60,75]. In our study, only half of the PCR-positive lungs showed macroscopic lesions (similar to the findings reported by Chiari et al., although another q-PCR protocol was used [28]) but the large majority of lungs did present histologic changes. In domestic pigs, the presence of histologic lung lesions in the absence of macroscopic changes also occurs, either in the case of subclinical infections [48,76] (when less virulent strains are involved [61,62] or after vaccination [77–79]), or when microscopic tissue damage remain after macroscopic lesions have healed (despite the fact that Mhyop may still be detected [12,60,80]).

BALT hyperplasia was the predominant histological feature observed in infected wild boar lungs. It is known from studies in pigs that infections with virulent strains of Mhyop are associated with high levels of interleukin-β and tumor necrosis factor in bronchial lavage fluids [61,72], and that these cytokines have a nonspecific mitogenic effect on lymphocytes, resulting in BALT hyperplasia [61,72,81,82]. In our study, BALT hyperplasia was present both in lungs without and with MaEPL, but we found significantly higher numbers of lymphoid follicles in hyperplastic BALT in late type MaEPL than in lungs without MaEPL. Since interleukin-β and tumor necrosis factor are mainly released at an early stage of the disease [83] wild boar lungs with only histologic lesions are likely at the beginning of the infectious process rather than in a healing phase. Nonetheless, so-called late type lesions may be related not only to the time factor but also to strain virulence [61,62,72]. Higher virulence may explain the higher number of lymphoid follicles (due to higher interleukin- β levels and higher mycoplasma proliferation), the association with neutrophil infiltration and the observed higher percentage of affected lung tissue in late type lesions than in early type lesions [61,72]. Yet, secondary bacterial infections may also contribute to the observed association between neutrophil infiltration and late type MaEPL, since bacterial infections are generally associated with neutrophilic infiltration and they represent a common finding in domestic pigs infected with Mhyop [1,2,45]. In addition, it has been reported that secondary infections contribute to the development of more severe EP lesions [2,84] and that secondary viral infections may also prolong the disease course [84,85]. Combined immuno-histochemical and Mhyop strain studies would be essential for better assessing the role of strain virulence in development of EP lesions in wild boar.

The impact of EP on the individual fitness and population dynamics of wild boar is unclear. We did not find an association between the presence of lesions or infection and poor body condition. The trend of wild boar population growth has been increasing over the past decades [32,86] including in Swiss regions with high Mhyop prevalence, suggesting that the EP impact on wild boar is negligible. Nevertheless, caution is warranted as our data on body condition were based on semiquantitative assessments in the fields and we lack information on non-anthropogenic causes of mortality in wild boar.

### Age as a risk factor

The observed infection pattern among age classes in the wild boar population, i.e. an increase of prevalence from an early age (piglets) to the middle age class (juveniles and subadults), followed by a decrease of prevalence in adults, has also been observed in a recent pathological and serological study in wild boar from Italy [28]. This pattern is similar to what is known from domestic pigs [7,87,88], in which all age classes are susceptible to infection with Mhyop [7,58,59,89] but post-weaning to fattening pigs display the highest prevalence of infection and lung lesions [2,6,90,91]. This is in part explained by the intrinsic immunity-related susceptibility to pathogens observed at this age [92]. Because juvenile and subadult wild boar are at least 6 months older than post-weaning pigs and are expected to be able to mount an immune response comparable to that of older age groups, the observed differences in prevalence among age classes in free-ranging wild boar must be due to other factors.

In domestic pigs, management factors such as age structure within a herd, intraspecific interactions and animal movements among herds play an important role in EP epidemiology. The housing of pigs of different ages in the same building is a risk factor for EP maintenance in a herd [88,90,93], and the infection rate with Mhyop in a herd increases exponentially when piglets are purchased from different farms and mixed during the fattening period [80,88,93]. In free-ranging wild boar, natural behaviors may lead to comparable situations and explain why infection is more frequent at a later age than in pigs. First, wild boar of all age classes live together, since a pack consists mainly of females of different ages and their offspring [94,95]. Second, exposure to other animals and the associated risk of intraspecific pathogen transmission increases around the age of late juvenile to the early subadult period, because offspring start dispersing [94–98] and solitary adult males join packs for the rut [94], which in turn may lead to pathogen and disease emergence [93,99] in susceptible wild boars.

Additionally, in domestic pigs large herd size (over 500 pigs) and high animal density have been accounted for the emergence of EP [7,80,93], while a low infection pressure leads to slower spread of Mhyop and longer subclinical expression (less to no coughing) at the herd level [90,100,101]. In wild boar, infection pressure is obviously expected to be much lower than in piggeries (with the exception of intensive management situations including fencing and feeding [23,102]) because packs of free-ranging wild boar are generally small [94,95,98] and even high densities such as that of our study unit A are incomparable to the dramatic densities reported in intensively managed pigs (<0.7m^2^/pig [93]). This may additionally contribute to the delayed age pattern of both EP lesions and Mhyop infection observed in free-ranging wild boar.

Finally, in domestic pigs stress is known to play a role in the epidemiology and pathogenesis of EP [2]. Changes such as dissolution of the maternal group, introduction into a new environment, diet modification and the arrival of new pen mates, have been shown to cause chronic stress in pigs [103,104]. Similar changes as well as their association to individual stress also occur in wild animals during the dispersal period [105–107]. Overall, both similarities and differences of the EP epidemiological pattern between domestic pigs and free-ranging wild boar may be explained by management issues and the hosts’ life history. This makes sense, knowing that wild boar and domestic pigs belong to the same species, and it has indeed been reported that intensification of wild boar management leads to increased occurrence of diseases typically associated with domestic pigs [102].

### Population and Environmental Factors

The marked differences in prevalence observed among geographical units resulted in the identification of two risk factors, i.e. wild boar density and the occurrence of EP outbreaks in domestic pigs. This is in agreement with observations in pigs [49].

Due to the lack of better methods of large scale data collection, hunting statistics are widely used for estimating wild boar population densities [23,108,109]. Data on population trends originating from hunting statistics are known to be poor but in unit A they correlate with those obtained with the capture-resight method [94] and our density estimation is in agreement with a previous report [19]. Since hunting pressure is expected to be comparable among our study units, we propose that our estimates are sufficiently reliable for our purpose. Thus, the high prevalence of infection in unit A (the highest of all units) may be related to the high wild boar population density in this area (also the highest of all units).

The occurrence of EP outbreaks in pigs was positively correlated with outdoor piggery density and negatively correlated with air temperature, as previously reported [49,93,110]. This also converges with the fact that the majority of outbreaks registered in this study were from domestic pig sources. According to our model, the occurrence of EP outbreaks in pigs plays a relevant role in the prevalence of Mhyop in wild boar. In agreement with this result, prevalence has increased in unit A since the last EP outbreak in 2007 (41%, n = 95 [31]; p-value = 0.0463) but seems now to be decreasing again. Similarly, prevalence seems to increase in unit B, where an EP outbreak recently occurred (2013). In contrast, a decrease of prevalence was observed from the first to the second hunting season in unit E (no EP outbreak since at least 1999) and prevalence has been stable to decreasing in unit C (last outbreak in 2011) and D (last outbreak in 2012). However, because a larger number of samples was collected during the second sampling round in unit E (2.5-fold higher than in the first) and this unit has the lowest Mhyop prevalence of all units, a sampling bias may have contributed to the overall decrease in prevalence. Nevertheless, it would be interesting to observe prevalence evolution at a local level over a longer period of time.

The geographical distribution of the three Mhyop types detected in this study revealed a clear difference between the northern and the southern wild boar populations. Thus, the northern population (units A-D, characterized by the occurrence of EP outbreaks in domestic pigs and a prominent overlap between wild boar and domestic pigs [32]) harbors mainly the ABC/REP-type, which is the one most commonly found in domestic pigs in Switzerland [14]. In contrast, the southern population (unit E, characterized by the absence of EP outbreaks and a very low density of outdoor piggeries) harbors mainly the ABC-type, less commonly detected in domestic pigs [14].

Overall, our data suggest that spillover of Mhyop from domestic pig to wild boar occurs and influences prevalence of infection in wild boar. Nevertheless, the fact that prevalence in unit A is now significantly higher than after the last outbreak in domestic pigs suggests that Mhyop has been maintained in the dense wild boar population of this region, and we cannot exclude the existence of parallel, independent domestic and sylvatic cycles.

### Assessing the Role of Wild Boar as a Reservoir for *M*. *hyopneumoniae*

The fact that wild boar is susceptible to the same Mhyop types as domestic pigs confirms that wild boar represent a potential source of infection for pigs. By definition, however, a reservoir not only maintains the pathogen, but it is also able to infect the target host population [111,112]. Therefore, to determine whether free-ranging wild boar are a reservoir for Mhyop, we should not only address the question as to whether they maintain the pathogen, but also as to whether they act as a (re)-infection source for domestic pigs. In this context, it is interesting to note that only one EP outbreak has been registered in unit A since 2004 [39] and that it has not been possible to demonstrate the role of wild boar as a source of infection [113]. Moreover, wild boar have been considered as a possible source of infection for only five out of 16 EP outbreaks registered in recent years in Switzerland—a hypothesis that has not yet been confirmed. As a matter of fact, close contacts between domestic pigs and free-ranging wild boar remain relatively rare events [22,32] and coughing, a main factor contributing to airborne transmission of Mhyop [8,44], is apparently not obvious in wild boar (it has not been mentioned by game-wardens and hunters, neither during general surveillance activities, nor during this targeted study). Airborne transmission of Mhyop between farms and over long distances [6,9,49] has been reported in areas of high piggery densities [49] but since animal density is much lower in the wild than within a herd of domestic pigs [93], infection pressure in nature must be lower than in piggeries, i.e. free-ranging wild boar likely represent a lower risk of airborne transmission of Mhyop over long distances. Furthermore, geographical features such as dense forests may have a filter effect on infectious aerosols [44].

Therefore, we propose that transmission of Mhyop from wild boar to domestic pig plays a minor role in the epidemiology of the disease in domestic pigs at the moment. It is important to keep in mind that, although wildlife is a recognized source of emerging pathogens, disease emergence is often the consequence of human activities such as animal transport between farms. This is well exemplified by multiple recent disease outbreaks including not only EP but also porcine reproductive respiratory syndrom [114], swine brucellosis [115] and bovine tuberculosis [116] in Swiss livestock. Nevertheless, this situation may change if wild boar occurrence, abundance and density continue to increase and the trend towards outdoor pig farming grows further [28,31,32]. High population densities together with an increased risk of contact is already a serious concern regarding other diseases involving wild boar and domestic pigs [22,86], which emphasizes the importance of improving biosecurity [32].

## Conclusion

This study demonstrates that Mhyop is widespread in the Swiss wild boar population (supporting our first study hypothesis) and that risk factors identified in domestic pigs (population density, young age and occurrence of EP outbreaks in domestic pig herds) are also significant for wild boar. However, we had to reject our second hypothesis when we found that infection in wild boar was often associated with lung lesions consistent with EP. The role of the wild boar as a reservoir remains unclear but our data indicates that spillover from domestic pigs is likely more frequent than spillback from wild boar. Strain analysis in both wildlife and livestock in areas with different infection patterns would be valuable to further address this question.

## Supporting Information

S1 Table Prevalence of infection per sampling unit and hunting season. Estimated prevalence of infection with *Mycoplasma hyopneumoniae* in wild boar is indicated for the five units A-E and for two consecutive hunting seasons. Prevalences are given in percent and followed by 95% confidence intervals in parentheses. P-values indicate the level of significance of the prevalence difference between the two hunting seasons.
(PDF)
