# Supplementary material for: Investigating the Role of Free-Ranging Wild Boar (Sus scrofa) in the Re-Emergence of Enzootic Pneumonia in Domestic Pig Herds: A Pathological, Prevalence and Risk-Factor Study
Source: PLoS One. 2015 Mar 6;10(3):e0119060. doi: 10.1371/journal.pone.0119060 (PMC4352045; doi:10.1371/journal.pone.0119060)
Supplement: S1 Table — Estimated prevalence of infection with Mycoplasma hyopneumoniae in wild boar is indicated for the five units A-E and for two consecutive hunting seasons. Prevalences are given in percent and followed by 95% confidence intervals in parentheses. P-values indicate the level of significance of the prevalence difference between the two hunting seasons. (PDF) [file pone.0119060.s001.pdf]

**Table S1. Prevalence of infection per sampling unit and hunting season.**

| Study unit | Hunting season 1 | Hunting season 2 | p-value |
|---|---|---|---|
| **A** | 60.4 (45.2-74.2) | 51.6 (40.9-62.2) | 0.3724 |
| **B** | 43.5 (30.9-56.7) | 47.6 (37.9-57.5) | 0.1207 |
| **C** | 20.0 (6.8-40.7) | 20.5 (9.2-36.4) | 1.000 |
| **D** | 42.1 (20.2-66.5) | 33.3 (21.0-47.4) | 0.5806 |
| **E** | 14.1 (7.9-22.5) | 6.2 (3.6-10.0) | 0.0313 |

Estimated prevalence of infection with *Mycoplasma hyopneumoniae* in wild boar is indicated for the five units A-E and for two consecutive hunting seasons. Prevalences are given in percent and followed by 95% confidence intervals in parentheses. P-values indicate the level of significance of the prevalence difference between the two hunting seasons.
